# Complete Transposition of the Great Arteries in the Pediatric Field: A Multimodality Imaging Approach

**DOI:** 10.3390/children11060626

**Published:** 2024-05-23

**Authors:** Sara Moscatelli, Martina Avesani, Nunzia Borrelli, Jolanda Sabatino, Valeria Pergola, Isabella Leo, Claudia Montanaro, Francesca Valeria Contini, Gabriella Gaudieri, Jessica Ielapi, Raffaella Motta, Marco Alfonso Merrone, Giovanni Di Salvo

**Affiliations:** 1 Centre for Inherited Cardiovascular Diseases, Great Ormond Street Hospital, London WC1N 3JH, UK; 2 Institute of Cardiovascular Sciences, University College London, London WC1E 6BT, UK; 3 Paediatric Cardiology Department, Royal Brompton and Harefield Hospitals, Guy’s and St. Thomas’ NHS Foundation Trust, London SW3 5NP, UK; 4 Division of Paediatric Cardiology, Department of Women and Children’s Health, University Hospital of Padua, 35128 Padua, Italy; 5 Adult Congenital Heart Disease Unit, AO Dei Colli-Monaldi Hospital, 80131 Naples, Italy; 6 Experimental and Clinical Medicine Department, University Magna Graecia of Catanzaro, 88100 Catanzaro, Italyi.leo@unicz.it (I.L.);; 7 Dipartimento di Scienze Cardio-Toraco-Vascolari e Sanità Pubblica, University Hospital of Padua, 35128 Padua, Italy; valeria.pergola@unipd.it (V.P.);; 8 Adult Congenital Heart Centre and National Centre for Pulmonary Hypertension, Royal Brompton Hospital, Guy’s and St. Thomas’ NHS Foundation Trust, London SW3 5NP, UK; 9 CMR Unit, Cardiology Department, Royal Brompton and Harefield Hospitals, Guy’s and St. Thomas’ NHS Foundation Trust, London SW3 5NP, UK; 10 National Heart and Lung Institute, Imperial College London, London SW3 6LY, UK; 11 Clinical Cardiology Unit, Department of Medical Sciences and Public Health, University Hospital of Cagliari, Strada Statale 554, Km 4.500, 09042 Monserrato, Italy; 12 Pediatric Cardiology and Congenital Heart Disease Unit, Brotzu Hospital, 09134 Cagliari, Italy; 13 Clinical Pathways and Epidemiology Unit, Bambino Gesù Children’s Hospital IRCCS, 00165 Rome, Italy; 14 Division of Cardiology and Cardio Lab, Department of Clinical Science and Translational Medicine, University of Rome Tor Vergata, 00133 Rome, Italy

**Keywords:** complete transposition of the great arteries, cardiovascular multimodal imaging, fetal echocardiography, pediatric cardiovascular magnetic resonance, cardiac computed tomography

## Abstract

The complete transposition of the great arteries (C-TGA) is a congenital cardiac anomaly characterized by the reversal of the main arteries. Early detection and precise management are crucial for optimal outcomes. This review emphasizes the integral role of multimodal imaging, including fetal echocardiography, transthoracic echocardiography (TTE), cardiovascular magnetic resonance (CMR), and cardiac computed tomography (CCT) in the diagnosis, treatment planning, and long-term follow-up of C-TGA. Fetal echocardiography plays a pivotal role in prenatal detection, enabling early intervention strategies. Despite technological advances, the detection rate varies, highlighting the need for improved screening protocols. TTE remains the cornerstone for initial diagnosis, surgical preparation, and postoperative evaluation, providing essential information on cardiac anatomy, ventricular function, and the presence of associated defects. CMR and CCT offer additional value in C-TGA assessment. CMR, free from ionizing radiation, provides detailed anatomical and functional insights from fetal life into adulthood, becoming increasingly important in evaluating complex cardiac structures and post-surgical outcomes. CCT, with its high-resolution imaging, is indispensable in delineating coronary anatomy and vascular structures, particularly when CMR is contraindicated or inconclusive. This review advocates for a comprehensive imaging approach, integrating TTE, CMR, and CCT to enhance diagnostic accuracy, guide therapeutic interventions, and monitor postoperative conditions in C-TGA patients. Such a multimodal strategy is vital for advancing patient care and improving long-term prognoses in this complex congenital heart disease.

## 1. Introduction 

Transposition of the great arteries (TGA) is one of the most prevalent cardiac congenital heart diseases (CHDs), accounting for 5–7% of all cardiac congenital malformations [1,2]. Without intervention, TGA could be life-threatening in the first weeks of life [2,3]. However, after effective medical and surgical treatments, early and midterm survival is nowadays achievable in the majority of cases and, with an overall good long-term prognosis [3]. 

Although the exact etiology and pathogenesis of TGA remains unknown [4], several theories have been postulated; the most accredited one involves an abnormal twisting of the pulmonary artery (PA) around the aorta during embryogenesis, with consequent misalignment of great vessels with the respective ventricular chamber [5,6]. An alternative theory suggests that an abnormal enlargement of the subaortic conus and resorption of the subpulmonary conus could be involved, resulting in the anterior and rightward positioning of the aorta in relation to the PA [7].

Two main types of TGA have been described: complete TGA (C-TGA) and congenitally corrected TGA (CC-TGA) [5]. 

With a prevalence ranging from 20.1 to 30.5 per 100,000 live births and a strong male preponderance (2:1) [2,4], C-TGA is a conotruncal abnormality characterized by atrioventricular concordance and ventriculoarterial discordance [5]. The aorta originates therefore from the morphological right ventricle (RV) while the PA originates from the morphological left ventricle (LV) [5]. The essential communication between the systemic and pulmonary circulations is typically maintained through an atrial septal defect (ASD). Other shunt sources such as ventricular septal defects (VSDs) or patent ductus arteriosus (PDA) do not maintain effective pulmonary blood flow and mainly function to augment the interatrial shunt by increasing left atrial pressure [5,8]. The diagnosis is usually fetal or neonatal and prompt surgical intervention is necessary for survival, with options including the atrial switch operation (AtrSO) [9,10], arterial switch operation (ASO) [11,12], and Rastelli [13] or Nikaidoh [14] operation in case of associated VSD. 

CC-TGA is a rarer anomaly, representing 1% of all forms of congenital heart disease [15] and is characterized by both atrioventricular and ventriculoarterial discordance [5,15,16,17,18,19]. For this review, we will focus on C-TGA, and we will not delve into the specifics of CC-TGA. For simplicity, from now on, we will refer to C-TGA as TGA throughout the manuscript.

Cardiac imaging therefore plays a central role in the initial assessment of TGA, aiding in the identification of both structural and functional anomalies. It is also key in assisting therapeutic management, providing a non-invasive evaluation of hemodynamic severity, the degree of associated intracardiac shunt, and the recognition of post-surgical complications. 

The aim of this review is to summarize the roles, advantages, and limitations of each imaging modality within the context of TGA. We have briefly summarized the use of these methods divided between prenatal, preoperative, and postoperative application (Table 1).

## 2. Fetal Echocardiogram in Complete Transposition of the Great Arteries

Prenatal diagnosis of TGA plays a pivotal role in modern pediatric cardiology, enabling early identification and planning for the management of this congenital heart defect. However, despite the advancements in fetal ultrasound screenings and fetal echocardiography, the rates of prenatal detection for TGA internationally remain relatively low, spanning from 25 to 40% [20,21].

**Figure 1:**
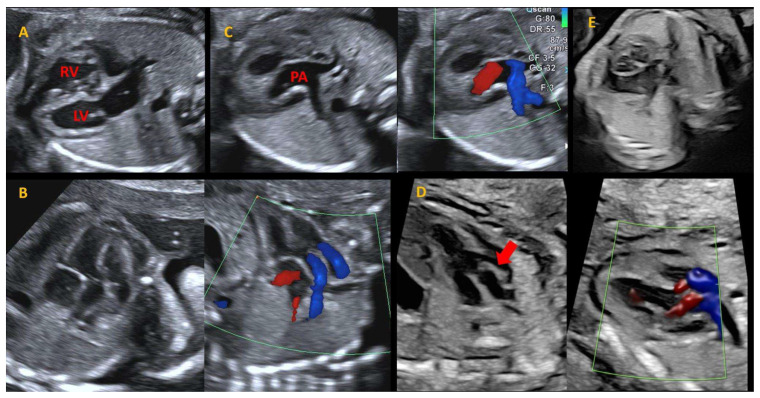
Fetal echocardiographic assessment in TGA through cranial sweep starting from 4CH going to the outflows. Panel (**A**): 4CH view, which appears normal in most fetuses with TGA; (**B**,**C**): outflow tracts’ assessment, showing parallel vessels arising from the 2 ventricles, with the posterior artery (PA), which bifurcates; (**D**): short-axis view, where both the semilunar valves appear in cross-sectional orientation; (**E**): the presence of a VSD should be investigated with 2D and color Doppler. In this panel, a mild posterior deviation of the conal septum is noted (red arrow).

One of the main reasons is that, until recently, the guidelines for obstetric ultrasound recommended the inclusion of the four-chamber (4CH) view in standard screening. Indeed, there was an optional evaluation of cardiac outflows, great arteries, and their relationship with the ventricles [22,23]. Nowadays, the assessment of left and right cardiac outflow tracts has been integrated as essential components of fetal echocardiographic 2-dimension (2D) imaging [24,25]. Consequently, the prenatal detection rates of TGA should improve. Usually, the diagnosis is confirmed by fetal echocardiography between 18 and 22 weeks of gestation following abnormal results on screening ultrasound. However, advances in imaging techniques now allow for the assessment of the fetal heart as early as 12 to 14 weeks’ gestation [26]. Timely recognition is fundamental as it enables healthcare providers to counsel expectant parents. They can plan appropriate interventions and arrange specialized care, thereby optimizing outcomes for infants born with TGA [27]. During a normal fetal scan, a cranial sweep from the 4CH view allows for the visualization of the left ventricle outflow tract (LVOT) and the aorta. More cranially, the right ventricle outflow tract (RVOT) and PA are visualized. Of note, in the normal heart, the great arteries cannot be seen in the same plane, meaning that they cross each other, and do not originate in parallel (Figure 1).

By contrast, in TGA, the great vessels arise in parallel from the ventricles. The origin of the main PA and its bifurcation from the LV and the aorta from the RV should be examined in two orthogonal planes, and any potential discrepancy in vessel size and flow acceleration should also be examined [28]. The three vessels and trachea view can raise the suspicion of TGA as well, demonstrating only a single large vessel (aorta) and the superior vena cava (SVC). Lastly, in the short-axis (SAX) view, both semilunar valves are seen in a cross-sectional orientation with the aorta usually anterior and to the right of the pulmonary artery.

Once the diagnosis of TGA is made, efforts should be made to identify anatomical features that can advocate the need for an urgent procedure after birth (i.e., Rashkind/atrial septostomy), modify the standard surgical approach (ASO), or alert the cardiologists about an increased risk of coronary abnormalities (i.e., side-by-side great vessels). VSD and pulmonary stenosis (PS) are the two most common associated cardiac findings in TGA. VSD is present in about 40% of cases and is usually a perimembranous outlet, but it can be located in any position and, if small, it can be missed during fetal scans due to equal ventricular pressure [29]. The presence of a posterior or anterior septal malalignment should also be investigated; indeed, they can cause respectively progressive PS, which is identified in about 30% of patients with VSD, or aortic root stenosis, and this can influence the postnatal management.

The identification of coronary artery (CA) origin in fetuses with TGA is of great importance. This information could help in counseling parents about potential variations in postnatal surgical techniques. 

Unfortunately, CA identification is still challenging for fetal cardiologists. Multiple factors, such as fetal position, gestational age, type of CA anatomy, and maternal habitus can affect the success of this evaluation. For many cases, it is necessary to repeat the examination to visualize all three CAs adequately. The early third trimester may represent the optimal gestational age for evaluating CA anatomy. Nevertheless, identifying the origin of CA presents challenges, particularly because the proximal course of the CA in certain types of anatomy closely aligns with the aortic root, making it difficult to visualize the CA flow or distinguish it from the aortic flow [30]. Recent data on 34 fetuses showed that coronary assessment was feasible in 41% of the cohort, with a higher chance of success after 25 weeks of gestation and when both short- and long-axis views were investigated [31].

Late-gestation imaging of the interatrial septum is also advisable to try to predict the need for a postnatal balloon (Rashkind) atrial septostomy and plan delivery in an environment where enlarging the interatrial septal defect is possible in case shunting proves to be insufficient. Several parameters have been investigated, including foramen ovale (FO) appearance, flow in the ductus arteriosus, maximal velocity of flow through the FO and pulmonary veins’ maximal velocity [32,33]. Among them, a flattened FO and an increased velocity in maximum pulmonary vein (PV) velocity (>41 cm/s) were associated with the need for a Rashkind procedure within the first 24 h postnatally and could be used prenatally to identify fetuses at risk for FO restriction [32,33].

## 3. Transthoracic Echocardiography in Complete Transposition of the Great Arteries

Transthoracic echocardiography (TTE) is a crucial diagnostic tool for the initial diagnosis, surgical planning, and late follow-up of patients with TGA. Indeed, for most neonates, TTE provides all the information required for a successful anatomical characterization and tailored surgical planning, hence obviating the need for further cardiovascular imaging modalities.

### 3.1. Preoperative Evaluation

Preoperative TTE protocol in patients with TGA is outlined in detail in Table 2. 

In the TTE evaluation of TGA, a segmental approach is fundamental. This method ensures a comprehensive assessment, including the spatial relationship between the aorta (Ao) and the pulmonary artery (PA). Typically, the aortic valve (AV) is positioned anteriorly and to the right of the pulmonary valve (PV), with the great vessels running parallel. In some cases, the AV may be located directly in front of the PV, side by side, or, less commonly, in an anterior–leftward position.

As previously emphasized, examining the interatrial septum is critical. This examination should identify any interatrial communications and assess the mixing efficiency between the systemic and pulmonary circulations. A thorough analysis includes evaluating the direction and velocity of flow across the septum [34]. In situations where the defect restricts adequate left-to-right shunting, necessary for oxygenated blood delivery to the systemic circulation, performing a balloon atrial septostomy might be warranted. The literature defines a restrictive interatrial communication by a mean gradient exceeding 8 mmHg, while a mean gradient below 3 mmHg is considered non-restrictive [35,36]. Both TTE and transesophageal echocardiography play a crucial role in guiding this intervention and evaluating its success.

**Figure 2:**
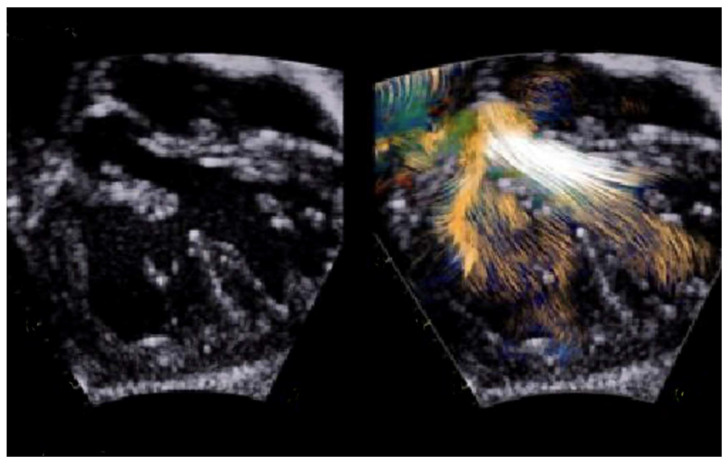
Laminar flow through the left outflow tract by blood speckle imaging.

Another key aspect of TTE is detecting any obstruction in the ventricular outflow tract, often due to deviation of the conal septum or valvar stenosis, as this may affect surgical planning [34]. Blood speckle imaging (Figure 1), a novel echocardiographic technique, enhances fluid dynamic analysis by overcoming the limitations associated with standard Doppler aliasing artifacts, thereby providing critical insights into distinguishing between genuine stenosis and volume mismatch (Figure 2) [37]. However, while this is a very promising technique, further evidence will be needed to validate its application in this setting.

The assessment of CA anatomy in TGA is critically important due to the anterior transposition of the aorta, which leads to significant variations in the origins and courses of the CA. These variations are particularly relevant because certain anatomical differences, such as the intramural course of the proximal CA, can complicate the ASO or lead to subsequent coronary events. The most frequent CA patterns observed in TGA, along with their approximate prevalence, include the usual pattern with the left coronary artery (LCA) originating from the left-facing sinus and the right coronary artery (RCA) from the posterior- and rightward-facing sinus; the origin of the circumflex artery (LCx) from the RCA; a single RCA emerging from the posterior-facing sinus; a single LCA from the left-facing sinus; inverted arteries, a configuration similar to a normal heart; inverted RCA and LCx; intramural LCA; and intramural RCA. Notably, intramural Cas in TGA often arise from the sinus facing the opposite direction and traverse within the aortic wall before exiting the adventitia, sharing a medium and lacking separate adventitial layers [34,35,36,37,38,39]. In addition to the commonly used apical four-chamber and high-parasternal short-axis views for identifying anomalous coronary origins, subxiphoid and modified apical five-chamber views can also be helpful. These views offer an additional perspective in evaluating coronary vasculature, especially in detecting a potential retro-pulmonary course of the LCx and revealing anomalous coronary origins [40]. 

Evaluating the interventricular septum is essential for identifying any ventricular communications, their anatomical types, and flow directions. The use of low Nyquist limits (≤60 cm/s) helps detect low-velocity shunts at the ventricular level, particularly since neonates’ right and left ventricular systolic pressures tend to be similar. While the decision to close a VSD is influenced by various factors, including surgical accessibility and visibility, generally, small VSDs (less than 3 mm) do not necessitate closure [41,42]. Lastly, Doppler echocardiography is invaluable for identifying PDA flow and its circulatory effects, excluding coarctation or interruption of the aortic arch, and pinpointing anomalies in the atrioventricular and semilunar valves that require surgical correction.

### 3.2. Postoperative Evaluation

For TGA, the ASO is the preferred method, providing both anatomical and physiological corrections. It involves cutting and switching the aorta and pulmonary artery positions (LeCompte maneuver) and relocating the coronary arteries to the newly positioned aorta. Certain conditions, like outflow tract obstructions or complex coronary patterns, might limit ASO’s applicability. When ASO is not viable, alternatives like the atrial switch procedure are used for cases with an intact ventricular septum, rerouting systemic and pulmonary venous blood at the atrial level. This method, however, has associated risks such as sinus node and ventricular dysfunction, leading to lower survival rates.

For TGA with ventricular septal defects, the Rastelli and REV procedures create a left-ventricle-to-aorta connection via an intraventricular tunnel. Rastelli uses an extracardiac conduit between the right ventricle and pulmonary artery, while REV employs the LeCompte maneuver for a direct right-ventricle-to-pulmonary-trunk connection. The Nikaidoh procedure, addressing complex anatomical issues, repositions the aortic root and coronary arteries, and corrects left ventricular outflow tract obstruction. Despite its complexity and higher reoperation risks, Nikaidoh offers improved physiological outcomes [42].

TTE is crucial for detecting post-surgical complications, although they are rare. Below, we highlight several key echocardiographic complications or critical factors that require attention [34]. Any patient with low-cardiac-output syndrome who has echocardiographic evidence of substantial LV dysfunction should be evaluated for CA stenosis. This is particularly important if regional motion abnormalities are identified. Recently, myocardial deformation imaging has emerged as a tool to assess regional wall motion abnormalities in this population. Indeed, several studies [43,44,45] have demonstrated reduced values of left ventricle global longitudinal strain in children who have undergone arterial switch operation with coronary reimplantation, underlining that select patients may be at greater risk of developing earlier ventricular dysfunction. In a study by Buendía-Fuentes et al. [46], reservoir, conduit, and contraction left atrial strain were found significantly reduced in patients with TGA after the arterial switch, highlighting a further possible risk of diastolic dysfunction in these patients. However, while these methods allow for the assessment of the risk of developing cardiac dysfunction resulting from CA stenosis, cardiac catheterization remains necessary to receive the diagnose in this scenario [4]. 

**Figure 3:**
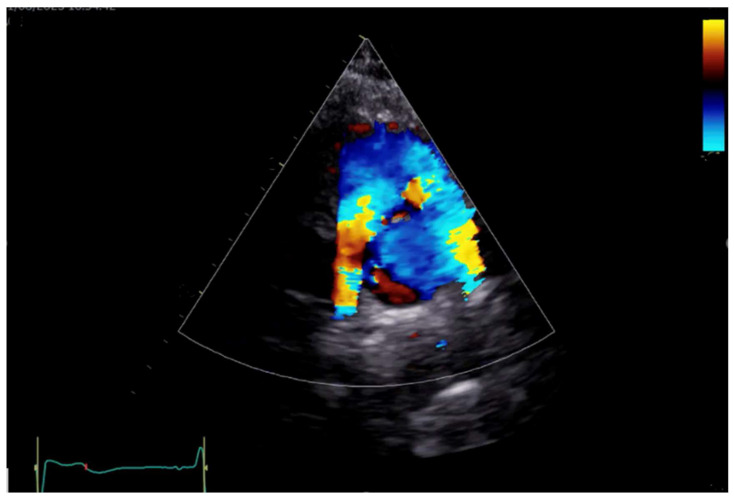
High-parasternal view showing color Doppler at level of pulmonary branches after LeCompte maneuver.

As a result of LeCompte maneuver’s deployment of the pulmonary artery anterior to the aorta, stenosis of the pulmonary branches is a common consequence. The short-axis high-parasternal imaging plane results, which are particularly valuable for a two-dimensional color Doppler and for spectral evaluations of pulmonary artery branches (Figure 3), are becoming more challenging from the adolescent period. A mild degree of flow acceleration (up to 2.5 m/s) is commonly observed and does not require further intervention [42]. Pulmonary outflow obstructions are usually observed in series at varying levels, including branches of the pulmonary artery and the suture site in the supravalvular pulmonary region [34].

The presence of aortic root dilatation with some grade of aortic insufficiency is a common complication, especially in patients with TGA and VSD, as opposed to TGA with intact ventricular septum [34,47]. Aortic root dilatation is progressive over time. A recent study reported a disproportional growth of neoaortic size in the first year after ASO, while the growth was reported as comparable to normal somatic growth, albeit with a higher z-score, in the years 2–18 after ASO [47]. 

### 3.3. Stress Echocardiography in Complete Transposition of the Great Arteries

Stress echocardiography could have a potential role in evaluating TGA in pediatric patients. The ASO necessitates coronary artery reimplantation, introducing a potential for complications such as tension, torsion, or the kinking of the vessels. Although coronary issues like stenosis, occlusion, or stretching are rare immediately post-surgery, they might emerge later during follow-up [48,49,50,51]. The basis of stress echocardiography lies in the observation by Tennant and Wiggers 80 years ago, which noted that coronary stenosis and myocardial ischemia result in myocardial wall motion abnormalities. Subsequent research has elaborated on the ischemic cascade, starting from subclinical metabolic changes to myocardial wall motion abnormalities and finally, angina symptoms. Stress echocardiography capitalizes on this knowledge to identify coronary artery disease (CAD) prior to the appearance of symptoms or ECG changes [48,49]. Consequently, as ASO is a substrate for coronary anomalies, stress echocardiography could identify myocardial ischemia before symptoms occur.

From a methodological point of view, stress echocardiography involves the induction of myocardial stress through exercise or pharmacological agents, followed by the assessment of cardiac function using echocardiographic imaging. Speckle-tracking in stress echocardiography enhances the assessment of myocardial mechanics, aiding in the detection of subtle abnormalities in patients with TGA [52]. It provides valuable insights into myocardial function under stress, contributing to risk stratification and therapeutic decision-making. Finally, three-dimensional echocardiography and contrast-enhanced imaging are increasingly being used for improving the sensitivity and specificity of stress echocardiography in TGA patients.

Stress echocardiography in TGA poses challenges, including imaging quality and interpretation complexities due to the altered cardiac anatomy and hemodynamics, as well as coronary anomalies. Furthermore, it is important to note that its use in this specific population has not yet been validated, and additional research and studies are needed to establish the utility and reliability of stress echocardiography in this clinical contest. Despite all this, a thorough evaluation combining clinical history, imaging data, and stress test outcomes could be helpful for accurate diagnosis and management planning in TGA patients.

## 4. Cardiovascular Magnetic Resonance (CMR) in Complete Transposition of the Great Arteries

CMR is an advanced imaging method. It provides a comprehensive view of thoracic and cardiac anatomy, function, flow, and tissue properties. Importantly, it does this without the use of ionizing radiation. This makes it extremely valuable for assessing conditions such as TGA, where detailed anatomical and functional insight is critical. Its non-invasive nature and safety profile make CMR a useful tool for patients of all ages, from fetal to adult age [53,54].

The main sequences used in CMR include cine balanced steady-state free precession (b-SSFP) sequences that are essential to define ventricular function, ventricular interdependency, and to assess great arteries and systemic vein stenosis visually, together with the presence of shunts at ventricular or atrial level and valvular regurgitation. All the above will be further assessed with phase contrast MRI, offering a defined quantification of shunt, regurgitation, and stenosis. Two-dimensional phase contrast MRI images are currently being integrated by the use of 4D flow sequences that offer various modalities of blood flow pathway visualization, providing unprecedented capabilities to understand blood flow changes using color-coded 3D multiplanar reformations, streamlines, and velocity vectors. Four-dimensional flow MRI allows for a retrospectively optimal assessment of any blood flow at any level and provides new advanced parameters, such as wall shear stress (WSS), kinetic energy loss, and pressure difference fields [55]. 

In addition, T1- and T2-weighted imaging for tissue characterization, providing essential information about myocardial edema and fat or scar content, and late gadolinium enhancement (LGE) identify myocardial fibrosis or scarring, important for prognosis and therapeutic planning [56]. Gadolinium and Ferumoxytol contrast agents are valid alternatives for angiography sequences that enable vasculature assessments [57]. Three-dimensional SSFP sequences offer high-resolution images of cardiac structures, enabling precise vascular assessment [56].

Despite its broad utility, CMR requires patient cooperation for breath-holding to ensure clear images; this can be challenging for young children. In such cases, the procedure might be performed under general anesthesia after clinical benefit–risk consultation. However, newer and faster techniques are being developed to reduce or eliminate the need for anesthesia in neonates and young children, improving CMR’s accessibility and safety for this vulnerable patient group [56].

Although its considerable utility in providing detailed diagnostic insights without ionizing radiation, MRI has specific contraindications and risks. It is not recommended for patients with non-MRI-compatible pacemakers, defibrillators, or certain metallic intracardiac devices due to the risk of artifacts or adverse interactions. However, the use of MRI-compatible devices has increased with the improvements in reducing metal artefacts [58]. Moreover, the use of contrast agents, while enhancing imaging clarity, may lead to allergic reactions or, in rare instances, nephrogenic systemic fibrosis in patients with compromised renal function. Thus, while MRI is a powerful diagnostic tool, its use must be judiciously considered against potential risks and contraindications [59].

### 4.1. Fetal CMR

Fetal CMR emerges as a powerful adjunct to echocardiography, especially in scenarios where traditional ultrasound faces limitations such as maternal obesity, oligohydramnios, unfavorable fetal positioning, and acoustic hindrances due to the fetal bony structure [60]. Fetal CMR could be performed with a 3 Tesla machine, which might achieve higher image quality permitting the visualization of small fetal cardiac structures, or with a 1.5 Tesla machine, which is safer and more accessible but with limitations in spatial resolution. During the acquisition, the mother should preferably lie in the supine decubitus position, or in the left lateral decubitus.

Fetal CMR protocol consists of static imaging, including b-SSFP sequences, and cine imaging with CINE-bSSFP sequences to investigate both anatomical and functional characteristics of the fetal heart [61]. Fetal CMR has been pivotal in elucidating detailed anatomical and functional insights into the fetal heart, offering precise definitions of the cardiac structure, connections, and size, thereby facilitating a deeper understanding of CHD [56,60]. Its application extends to diagnosing vascular anomalies with a high degree of accuracy, often surpassing echocardiography, and providing invaluable information for the management and intervention in cases like TGA [62]. The fetal CMR is a promising tool for planning interventions such as balloon atrial septostomy in utero to mitigate severe hypoxemia after birth, showcasing its critical role in guiding therapeutic decisions and assessing the efficacy of interventions to ensure a smoother transition from fetal to neonatal circulation [63].

### 4.2. CMR in Neonatal and Pediatric Life

CMR in the neonatal and pediatric population, especially for conditions like TGA, requires careful consideration due to the long scan times and the need for patient cooperation. Traditionally, CMR has been challenging in children under 8 years old due to the necessity for breath-holding, often necessitating general anesthesia for younger or neonatal patients (56). However, the advent of four-dimensional (4D) flow MRI technology offers a promising shift toward nonsedated, free-breathing acquisition protocols. Innovations like the “feed and wrap” technique [64], demonstrate the feasibility of sedation-free neonatal MRI, providing flow and volume quantifications that align closely with traditional 2D phase contrast methods. This development increases the introduction of MRI in neonates and pediatric patients at any stage of clinical treatment [34,65].

### 4.3. Preoperative

Despite these technological advancements, CMR is infrequently utilized for the preoperative evaluation of infants with TGA, as echocardiography suffices for surgical planning by detailing intracardiac anatomy and ventricular outflow tract obstruction mechanisms [66]. CMR’s role is often reserved for assessing thoracic vessels when echocardiography is inconclusive. Although CMR can precisely quantify LV mass, volume, and systolic function, criteria for determining adequate ventricular preparation for surgery remain to be clearly established. This highlights the specific, yet evolving role of CMR in the comprehensive assessment and management of TGA in neonatal and pediatric patients before surgery [34,56,65].

### 4.4. Postoperative

CMR imaging plays a pivotal role in the postoperative evaluation and management of patients with C-TGA across various surgical interventions, offering a comprehensive and non-invasive modality that complements echocardiography [34,56,58,65]. 

In the context of the ASO, CMR emerges as an indispensable tool, particularly when increasing patient size and postoperative scar tissue limit the effectiveness of echocardiography. It excels in detecting branch PAs’ stenosis and flow differential [66], accurately quantifying ventricular parameters, and resolving uncertainties regarding the severity of valve regurgitation [67]. Furthermore, CMR’s high-resolution imaging capabilities enable the precise assessment of proximal CAs and their relation to surrounding structures, aiding in the diagnosis of inducible CA ischemia and myocardial infarction through stress perfusion and late LGE techniques [68,69,70].

**Figure 4:**
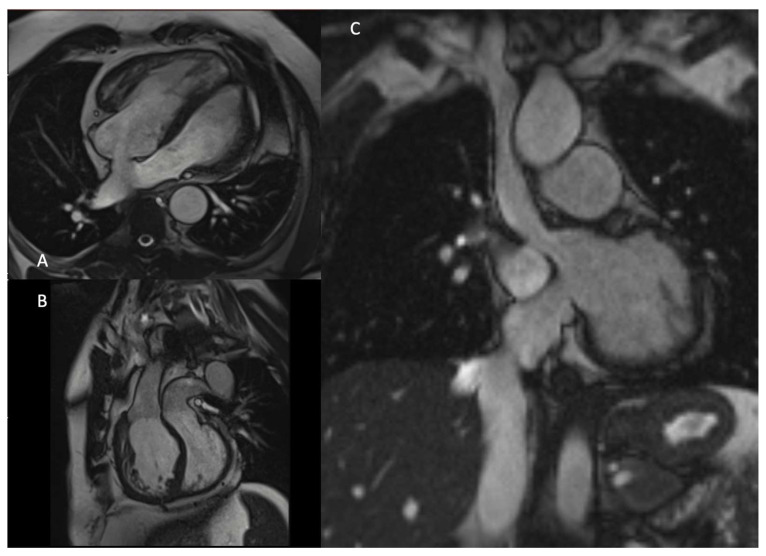
Male patient with TGA after ArtSO-Mustard operation. (**A**) A 4CH view with patent pulmonary venous pathway; (**B**) aorta and PA running in parallel with aorta emerging from RV and PA form LV; (**C**) patent systems’ venous baffle with unobstructed superior vena cava and inferior vena cava.

Nowadays, AtrSO is a far less common surgical option, but when performed, CMR assumes a central role in imaging surveillance, especially in assessing the systemic RV, which can be challenging with echocardiography due to its position and complex shape (Figure 4). It accurately and reproducibly measures the ventricle’s volume, mass, and ejection fraction and assesses systemic and pulmonary venous baffle pathways for obstructions or leaks. The detection of right ventricular focal myocardial fibrosis through LGE is associated with adverse outcomes, highlighting CMR’s prognostic significance [71], together with the detection of baffle obstructions [72].

In patients who have undergone the Rastelli or Nikaidoh procedures, CMR offers unparalleled imaging of the pathway of the outflows from the left ventricle to the aortic valve and from the right ventricle to the pulmonary artery via a conduit. It assesses for obstructions, estimates gradients across stenoses, quantifies valve regurgitation, and evaluates the impact of residual VSDs. This comprehensive assessment is crucial for determining the need for surgical conduit replacement or catheter-based interventions [73].

CMR 3D models offer a distinct advantage for surgical planning, providing a comprehensive visualization of the anatomical structures together with precise measurements of cardiac dimensions and volumes [74,75].

Overall, CMR stands out for its ability to provide detailed anatomical and functional insights after surgical corrections in TGA, significantly impacting clinical decision-making, management strategies, and long-term surveillance of these patients.

## 5. Cardiac Computed Tomography (CCT) in Complete Transposition of the Great Arteries

In recent years, the evaluation of congenital heart diseases in pediatric patients has experienced a significant transformation, with cardiac computed tomography (CCT) playing an increasingly important role [4,76]. Technological advancements, such as enhanced spatial and temporal resolutions, the rapid acquisition of isotropic volumetric data, and reduced radiation oses, have significantly improved diagnostic accuracy [77]. Dual-source and wide-detector scanners, among other innovations, contribute to improved temporal resolution and reduced motion artifacts, which are particularly advantageous in neonates and infants [78].

A key factor contributing to the growing significance of CCT is the substantial reduction in radiation exposure and the diminishing need for sedation. State-of-the-art scanners rapidly acquire datasets, eliminating the necessity for prolonged breath-holding and sedation in neonates and infants, thereby enhancing patient safety [79]. The utilization of 320- or 640-section 16 cm detectors in newborns and young children has further expanded *z*-axis coverage and accelerated image acquisition, resulting in a remarkable 60–80% reduction in radiation exposure [80]. The reduction in or elimination of overlapping helical imaging contributes to an overall risk mitigation strategy. For older-generation scanners with longer acquisition times (>10 s), sedation or anesthesia may still be required in children. Recent developments have reduced radiation exposure, achieving <5 mSv for a combined CCT coronary, pulmonary, and aortic angiogram. Automated dose modulation and iterative reconstruction algorithms further reduce radiation doses while maintaining diagnostic image quality [77].

The increasing adoption of CCT is evident in temporal trends, surpassing the rise in CMR use [81]. However, despite these advancements, challenges persist in aligning clinical practice with appropriateness criteria (AUC) for CHD imaging, which may not fully encompass CCT’s expanding role, especially in procedural planning for defects like tetralogy of Fallot or TGA [82]. Typically, 2 mL/kg of a contrast agent is administered, diluted if necessary, with injection rates adjusted to maintain a 15–25 s bolus duration, not exceeding the total fluid limits of 10 mL/kg. 

Protocols may require individual modifications. Weight-limited contrast doses and challenges like tachycardia are considered. Often, biphasic injection protocols are used, deploying a neat contrast bolus followed by a saline chaser. Power injectors are preferred for the precise control of injection rates [81].

### 5.1. Preoperative Imaging

In TGA, preoperative cardiac CT is employed to delineate coronary anatomy and examine complex vascular structures in cases involving heterotaxy syndrome. The preoperative checklist encompasses the assessment of great vessel origin and relationships, interatrial and interventricular communication, outflow tract obstructions, semilunar valve stenosis, ductal arteriosus status, coronary artery anomalies, and aortic arch conditions. Despite significant advancements, the imaging of systemic and pulmonary venous vasculature remains dependent on the timing of contrast administration. This method can sometimes miss critical anatomical details that are not enhanced during the acquisition phase [78].

The robust capability of cardiac CT resides in its ability to generate detailed 3D reconstructions, enabling the precise identification and characterization of complex coronary anatomies. The significance of coronary artery nomenclature becomes paramount in cases of TGA, shaping treatment decisions and surgical planning [83]. 

Three-dimensional reconstructions form the foundation for 3D modeling, which is increasingly being recognized as a valuable tool in surgical preparation [74,75].

**Figure 5:**
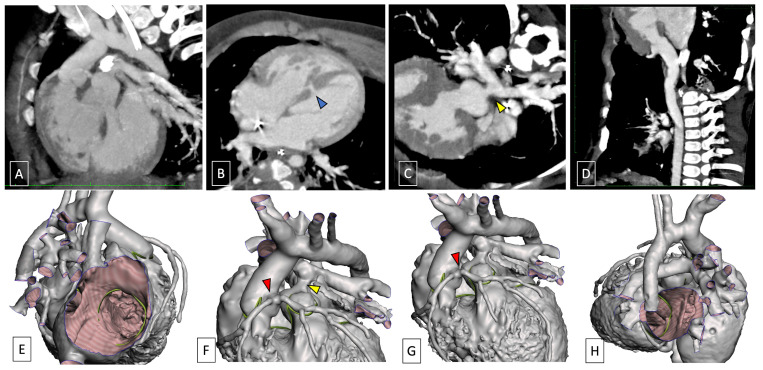
A 4-month-old infant, scanned in sedation and free-breathing at 96 bpm, with TGA-type DORV and VSD in (**A**) and (**B**) MPR view-blue arrow; (**E**) and (**H**) in VRT reconstruction and s/p pulmonary artery banding; (**C**) curved MPR view (aortic coarctation s/p aortic coarctation treatment—yellow arrow); (**D**) curved MPR view (anomalous coronary arteries with common origins of both right and left coronary arteries from the same sinus of Valsalva (**F**,**G**—**red arrow**) in VRT reconstruction). Yellow arrows indicate pulmonary artery banding.

In TGA, where the aorta and pulmonary artery undergo transposition, the Leiden classification assigns numerical designations to sinuses crucial for surgical procedures. The right posterior facing sinus (Leiden 2) typically gives rise to the RCA, while the left anterior facing sinus (Leiden 1) contributes to the LAD and LCx [84]. This aids in identifying potential complications and guiding therapeutic interventions [83,84]. Beyond sinus relationships, meticulous reporting includes details of coronary arteries’ origins within or above a sinus and the notation of intramural segments (Figure 5). Rare occurrences, such as an intramural segment coursing within the aortic wall, pose inherent risks [83].

### 5.2. Postoperative Imaging

Following AtrSO procedures, the imaging targets encompass the status of systemic or pulmonary venous baffles, residual ventricular septal defects (VSDs), main or branch pulmonary arteries (PAs), and potential obstructions in the right or left outflow tracts. An evaluation of the systemic right ventricular (RV) functional status is also crucial, particularly due to the complex three-dimensional structure of venous pathways, and is challenging to assess with echocardiography [78].

The common site for narrowing in the systemic venous pathway is typically at the entrance of the distal superior limb into the right atrium. Computed tomography (CT) proves advantageous in visualizing both systemic and pulmonary venous pathways, with CT being the preferred method for detecting and treating anatomic stenoses, often addressed through stent placements. In cases where stent restenosis is suspected, CT is the imaging modality of choice [81].

Post-ASO, patients often require pacing and defibrillator leads. CT is instrumental in observing these leads, and in instances of repeat electrophysiologic intervention, such as the placement of biventricular pacing leads, CT is the preferred modality for evaluating coronary venous anatomy. Moreover, CT serves as a valuable tool for calculating ventricular volumes, mass, and ejection fraction, especially when echocardiography is insufficient, and CMR is not feasible [82].

The objectives of postoperative imaging in ASO include assessing the relationships of great vessels, the integrity of reimplanted coronaries, neoaortic root dilatation, PA stenosis, and aortopulmonary collaterals. CT, reserved for patients with contraindications for cardiac MRI, offers a comprehensive two-dimensional and three-dimensional anatomical assessment of the neopulmonary root, neoaortic root, branch PAs, and reimplanted coronary arteries. Notably, coronary artery lesions, including ostial stenosis, kinking, anomalous course, and atherosclerotic disease, are common in post-ASO patients, and CTA facilitates their detailed evaluation [76].

For those undergoing angioplasty for PA stenosis, CT emerges as the ideal method to assess PA stents due to potential artifacts with cardiac MRI. CT plays a crucial role in visualizing three-dimensional structures like systemic and pulmonary venous pathways, aiding in the assessment of potential stenosis or thrombosis. Furthermore, in a subset of ASO patients, the evaluation of gothic aortic arch poses challenges, impacting cardiac mechanics and exercise capacity [85,86].

## 6. Conclusions

Multimodality imaging is essential for the comprehensive diagnosis and management of TGA, both before and after surgery. Imaging techniques are instrumental in identifying cardiac anatomical or functional abnormalities, assisting in treatment decisions, and guiding the timing of interventions. In addition, a multimodality imaging approach is crucial during the follow-up phase to detect complications and assess the potential need for further intervention.

Various imaging techniques play established roles in this context. The diagnosis of TGA may be achievable during the fetal period through a careful echocardiographic assessment, providing an opportunity for prenatal counseling and planning for prenatal/neonatal management [2,56].

Echocardiography represents the primary imaging modality for both the diagnosis and follow-up, given its widespread availability, low cost, and absence of radiation exposure [2]. It provides valuable insights into structural and functional abnormalities, as well as the hemodynamic status of patients [36]. However, it may not be the optimal choice for complex cases or instances with poor acoustic windows, particularly for right ventricle assessment.

In such cases, CMR represents the preferred imaging modality, offering detailed information to define complex anatomy, assess myocardial and valvular function, and detect extracardiac abnormalities. Additionally, it also uniquely provides myocardial tissue characterization [87,88]. However, CMR is contraindicated in patients with certain metallic devices or claustrophobic disturbances, and its relatively longer duration may limit its use, particularly in children, sometimes requiring anesthesia to complete the examination [88].

Finally, CCT is employed when contraindications to or artifacts in CMR are present [77]. CCT allows for the assessment of cardiac and coronary anatomy and provides important insights into ventricular function [88]. While CCT offers similar data to CMR, it lacks hemodynamic information and exposes patients to ionizing radiations and contrast material administration. Furthermore, the application of CCT and CMR 3D models into the assessment of patient TGA holds great promise for optimizing the management of this specific population [74,75].

Certainly, the selection of an imaging modality is influenced by various factors, primarily driven by the specific clinical question, the current status of the disease, and the presence of contraindications. In addition, practical considerations such as local availability and the level of expertise of the medical professionals involved play a crucial role in the decision-making process [36]. Each modality has its strengths and limitations, and the optimal choice depends on a careful evaluation of these factors to ensure the most effective and safe diagnostic and management approach for the patient.

## Figures and Tables

**Table 1:** Summary of the use of echocardiography, cardiovascular magnetic resonance, and cardiac computed tomography in the prenatal, preoperative, and postoperative periods.

	Prenatal	Preoperative	Postoperative
**Echocardiography**	Early diagnosis Planning delivery and fetal or early intervention/delivery Outflow tracts’ assessment CA origins’ assessment	Evaluation of mixing between the systemic and pulmonary circulations: VSDs, PDA, ASDs Proximal CAs’ anatomy assessment Outflow tracts’ assessments (valvar stenosis/regurgitation, outflow tract obstructions) Intervention planning	Postoperative complications (ventricular disfunction, PAs stenosis but with limited vitalization from adolescence) Stress echocardiography: evaluation of CA reimplantation/inducible myocardial ischemia
**Cardiovascular Magnetic Resonance**	An alternative to fetal echocardiography in scenarios where traditional ultrasound faces limitations Still considered a research method Diagnosis of vascular anomalies with a high degree of accuracy Support in planning fetal or early intervention/delivery	Limited use due to need of high patient compliance Detailed definition of cardiac anatomy, ventricular volumes, function, and vascular structures 3D modeling	Detailed definition of cardiac anatomy, ventricular volumes, function, and vascular structures Pivotal role in postoperative evaluation and management (e.g., baffle obstructions, PA stenosis, PA flow distribution) Tissue characterization (myocardial ischemia) Origin and proximal part of CAs Stress CMR: evaluation of CA reimplantation/inducible myocardial ischemia 3D modeling
**Cardiac Computed Tomography**	Contraindicated due to radiation exposure	Cardiac anatomy and vascular structure assessment Gold standards for CA anatomy assessment 3D modeling	Vascular structure assessment Assessment of region where stents are in place Evaluation of CA anatomy post-reimplantation 3D modeling

**Table 2:** Echocardiographic assessment in patients with TGA.

Preoperative Assessment	
Spatial relationship between the aorta and the pulmonary artery	Subxiphoid frontal Subxiphoid sagittal Parasternal long axis
Presence and size of the atrial septal defect	Subxiphoid frontal
Presence, location, number, and size of VSD	Subxiphoid sagittal Apical five chamber Parasternal short axis
AV valve morphology, function, and abnormalities	Subxiphoid sagittal Apical four and five chamber
Outflow tract obstruction	Subxiphoid sagittal Apical five chamber Parasternal long axis
Coronary artery anatomy and anomalies	High parasternal short axis Apical four chamber Subxiphoid
Aortic arch anatomy and sideness and PDA	Suprasternal sagital
**Postoperative Assessment**	
Residual ASD	Subxiphoid frontal
Residual VSD	Subxiphoid frontal Subxiphoid sagittal Parasternal short axis
Ventricular function, size, regional wall motion, AV valve function	Apical four chamber Parasternal long axis Parasternal short axis
Outflow tract obstruction, neoaortic root dilation, semilunar valve regurgitation/stenosis, supravalvar stenosis	Subxiphoid frontal Subxiphoid sagittal Apical five chamber Parasternal long axis Parasternal short axis
Branch pulmonary arteries stenosis	High parasternal plane
Residual arch obstruction or residual PDA	Suprasternal sagittal Subxiphoid sagittal

## Abbreviations

ASD	atrial septal defect
ASO	arterial switch operation
AtrSO	atrial switch operation
AV	aortic valve
b-SSFP	balanced steady-state free precession
CA	coronary arteries
CAD	coronary artery disease
CCT	cardiac computed tomography
CTGA	complete TGA
CCTGA	congenital corrected TGA/L-TGA
CFR	coronary flow reserve
CHD	congenital heart disease
CX	circumflex artery
DORV	double outlet right ventricle
FFSE	fast spin echo sequences
GAA	gothic aortic arch
GBCA	gadolinium-based contrast agents
HASTE	(half-Fourier acquisition single shot)
LAD	left anterior descending artery
LCA	left coronary artery
LCx	left circumflex
LGE	late gadolinium enhancement
LV	left ventricle
LVOT	left ventricular outflow tract
LVOTO	left ventricular outflow obstruction
MRI	magnetic resonance imaging
PA	pulmonary artery
PDA	patent ductus arteriosus
PFO	patent foramen ovale
PS	pulmonary stenosis
RA	right atrium
RCA	right coronary artery
RV	right ventricle
RVOT	right ventricular outflow tract
SAX	short axis
SVC	superior vena cava
TGA	transposition great arteries
TTE	transthoracic echocardiography
US	ultrasound
VSD	ventricular septal defect
WSS	wall shear stress
PS	pulmonary stenosis
PV	pulmonary valve
PVs	pulmonary veins
2D	two dimension
4CH	four chamber

## References

[1] Samánek M., Slavík Z., Zborilová B., Hrobonová V., Vorísková M., Skovránek J. (1989). Prevalence, Treatment, and Outcome of Heart Disease in Live-Born Children: A Prospective Analysis of 91,823 Live-Born Children. Pediatr. Cardiol..

[2] Sarris G.E., Balmer C., Bonou P., Comas J.V., da Cruz E., Chiara L.D., Di Donato R.M., Fragata J., Jokinen T.E., Kirvassilis G. (2017). Clinical Guidelines for the Management of Patients with Transposition of the Great Arteries with Intact Ventricular Septum. Eur. J. Cardio-Thorac. Surg. Off. J. Eur. Assoc. Cardio-Thorac. Surg..

[3] Wernovsky G. (2016). Transposition of the Great Arteries and Common Variants. Pediatr. Crit. Care Med. J. Soc. Crit. Care Med. World Fed. Pediatr. Intensive Crit. Care Soc..

[4] Baumgartner H., De Backer J., Babu-Narayan S.V., Budts W., Chessa M., Diller G.-P., Lung B., Kluin J., Lang I.M., Meijboom F. (2021). 2020 ESC Guidelines for the Management of Adult Congenital Heart Disease: The Task Force for the Management of Adult Congenital Heart Disease of the European Society of Cardiology (ESC). Endorsed by: Association for European Paediatric and Congenital Cardiology (AEPC), International Society for Adult Congenital Heart Disease (ISACHD). Eur. Heart J..

[5] Warnes C.A. (2006). Transposition of the Great Arteries. Circulation.

[6] Salerno N., Panuccio G., Sabatino J., Leo I., Torella M., Sorrentino S., De Rosa S., Torella D. (2023). Cellular and Molecular Mechanisms Underlying Tricuspid Valve Development and Disease. J. Clin. Med..

[7] Van Praagh R., Van Praagh S. (1966). Isolated Ventricular Inversion. A Consideration of the Morphogenesis, Definition and Diagnosis of Nontransposed and Transposed Great Arteries. Am. J. Cardiol..

[8] Ashworth M., Al Adnani M., Sebire N.J. (2006). Neonatal Death Due to Transposition in Association with Premature Closure of the Oval Foramen. Cardiol. Young.

[9] Senning A. (1959). Surgical Correction of Transposition of the Great Vessels. Surgery.

[10] Mustard W.T. (1964). Successful two-stage correction of transposition of the great vessels. Surgery.

[11] Yacoub M.H., Radley-Smith R., Hilton C.J. (1976). Anatomical Correction of Complete Transposition of the Great Arteries and Ventricular Septal Defect in Infancy. Br. Med. J..

[12] Jatene A.D., Fontes V.F., Paulista P.P., de Souza L.C., Neger F., Galantier M., Souza J.E. (1975). Successful Anatomic Correction of Transposition of the Great Vessels. A Preliminary Report. Arq. Bras. Cardiol..

[13] Rastelli G.C., Wallace R.B., Ongley P.A. (1969). Complete Repair of Transposition of the Great Arteries with Pulmonary Stenosis. A Review and Report of a Case Corrected by Using a New Surgical Technique. Circulation.

[14] Nikaidoh H. (1984). Aortic Translocation and Biventricular Outflow Tract Reconstruction. A New Surgical Repair for Transposition of the Great Arteries Associated with Ventricular Septal Defect and Pulmonary Stenosis. J. Thorac. Cardiovasc. Surg..

[15] Ravishankar C. (2016). L-Transposition of the Great Arteries. Pediatr. Crit. Care Med. J. Soc. Crit. Care Med. World Fed. Pediatr. Intensive Crit. Care Soc..

[16] Warnes C.A. (1996). Congenitally Corrected Transposition: The Uncorrected Misnomer. J. Am. Coll. Cardiol..

[17] Graham T.P., Bernard Y.D., Mellen B.G., Celermajer D., Baumgartner H., Cetta F., Connolly H.M., Davidson W.R., Dellborg M., Foster E. (2000). Long-Term Outcome in Congenitally Corrected Transposition of the Great Arteries: A Multi-Institutional Study. J. Am. Coll. Cardiol..

[18] Kutty S., Danford D.A., Diller G.-P., Tutarel O. (2018). Contemporary Management and Outcomes in Congenitally Corrected Transposition of the Great Arteries. Heart Br. Card. Soc..

[19] Friedberg D.Z., Nadas A.S. (1970). Clinical Profile of Patients with Congenital Corrected Transposition of the Great Arteries. A Study of 60 Cases. N. Engl. J. Med..

[20] Marek J., Tomek V., Skovránek J., Povysilová V., Samánek M. (2011). Prenatal Ultrasound Screening of Congenital Heart Disease in an Unselected National Population: A 21-Year Experience. Heart Br. Card. Soc..

[21] van Velzen C.L., Haak M.C., Reijnders G., Rijlaarsdam M.E.B., Bax C.J., Pajkrt E., Hruda J., Galindo-Garre F., Bilardo C.M., de Groot C.J.M. (2015). Prenatal Detection of Transposition of the Great Arteries Reduces Mortality and Morbidity. Ultrasound Obstet. Gynecol. Off. J. Int. Soc. Ultrasound Obstet. Gynecol..

[22] American Institute of Ultrasound in Medicine (2010). AIUM Practice Guideline for the Performance of Obstetric Ultrasound Examinations. J. Ultrasound Med. Off. J. Am. Inst. Ultrasound Med..

[23] Salomon L.J., Alfirevic Z., Berghella V., Bilardo C., Hernandez-Andrade E., Johnsen S.L., Kalache K., Leung K.-Y., Malinger G., Munoz H. (2011). Practice Guidelines for Performance of the Routine Mid-Trimester Fetal Ultrasound Scan. Ultrasound Obstet. Gynecol. Off. J. Int. Soc. Ultrasound Obstet. Gynecol..

[24] Moon-Grady A.J., Donofrio M.T., Gelehrter S., Hornberger L., Kreeger J., Lee W., Michelfelder E., Morris S.A., Peyvandi S., Pinto N.M. (2023). Guidelines and Recommendations for Performance of the Fetal Echocardiogram: An Update from the American Society of Echocardiography. J. Am. Soc. Echocardiogr..

[25] Carvalho J., Allan L., Chaoui R., Copel J., DeVore G., Hecher K., Lee W., Munoz H., Paladini D., Tutschek B. (2013). ISUOG Practice Guidelines (Updated): Sonographic Screening Examination of the Fetal Heart. Ultrasound Obstet. Gynecol. Off. J. Int. Soc. Ultrasound Obstet. Gynecol..

[26] Huggon I.C., Ghi T., Cook A.C., Zosmer N., Allan L.D., Nicolaides K.H. (2002). Fetal Cardiac Abnormalities Identified Prior to 14 Weeks’ Gestation. Ultrasound Obstet. Gynecol. Off. J. Int. Soc. Ultrasound Obstet. Gynecol..

[27] Bonnet D., Coltri A., Butera G., Fermont L., Le Bidois J., Kachaner J., Sidi D. (1999). Detection of Transposition of the Great Arteries in Fetuses Reduces Neonatal Morbidity and Mortality. Circulation.

[28] Carvalho J.S., Axt-Fliedner R., Chaoui R., Copel J.A., Cuneo B.F., Goff D., Gordin Kopylov L., Hecher K., Lee W., Moon-Grady A.J. (2023). ISUOG Practice Guidelines (Updated): Fetal Cardiac Screening. Ultrasound Obstet. Gynecol. Off. J. Int. Soc. Ultrasound Obstet. Gynecol..

[29] Allen H.D., Driscoll D.J., Shaddy R.E., Feltes T.F. (2013). Moss & Adams’ Heart Disease in Infants, Children, and Adolescents: Including the Fetus and Young Adult.

[30] Kaji T., Hayabuchi Y., Maeda K., Irahara S.N.M. (2017). Prenatal assessment of coronary artery anatomy using color Doppler in cases of D-transposition of the great arteries: Case reports. J. Obstet. Gynaecol. Res..

[31] Haligheri G., Patel C.R., Komarlu R. (2021). Prenatal Delineation of Coronary Anatomy in Dextro-Transposition of Great Arteries. J. Cardiovasc. Echogr..

[32] Wilson A.D., Rao P.S., Aeschlimann S. (1990). Normal Fetal Foramen Flap and Transatrial Doppler Velocity Pattern. J. Am. Soc. Echocardiogr. Off. Publ. Am. Soc. Echocardiogr..

[33] Słodki M., Axt-Fliedner R., Zych-Krekora K., Wolter A., Kawecki A., Enzensberger C., Gulczyńska E., Respondek-Liberska M. (2018). International Prenatal Cardiology Collaboration Group New Method to Predict Need for Rashkind Procedure in Fetuses with Dextro-Transposition of the Great Arteries. Ultrasound Obstet. Gynecol. Off. J. Int. Soc. Ultrasound Obstet. Gynecol..

[34] Cohen M.S., Eidem B.W., Cetta F., Fogel M.A., Frommelt P.C., Ganame J., Han B.K., Kimball T.R., Johnson R.K., Mertens L. (2016). Multimodality Imaging Guidelines of Patients with Transposition of the Great Arteries: A Report from the American Society of Echocardiography Developed in Collaboration with the Society for Cardiovascular Magnetic Resonance and the Society of Cardiovascular Computed Tomography. J. Am. Soc. Echocardiogr..

[35] Graziano J.N., Heidelberger K.P., Ensing G.J., Gomez C.A., Ludomirsky A. (2002). The Influence of a Restrictive Atrial Septal Defect on Pulmonary Vascular Morphology in Patients with Hypoplastic Left Heart Syndrome. Pediatr. Cardiol..

[36] Hoque T., Richmond M., Vincent J.A., Bacha E., Torres A. (2013). Current Outcomes of Hypoplastic Left Heart Syndrome with Restrictive Atrial Septum: A Single-Center Experience. Pediatr. Cardiol..

[37] Borrelli N., Avesani M., Sabatino J., Ibrahim A., Josen M., Paredes J., Di Salvo G. (2021). Blood Speckle Imaging: A New Echocardiographic Approach to Study Fluid Dynamics in Congenital Heart Disease. Int. J. Cardiol. Congenit. Heart Dis..

[38] Cohen M.S., Mertens L.L. (2019). Educational Series in Congenital Heart Disease: Echocardiographic Assessment of Transposition of the Great Arteries and Congenitally Corrected Transposition of the Great Arteries. Echo Res. Pract..

[39] Baraona F., Valente A.M., Porayette P., Pluchinotta F.R., Sanders S.P. (2012). Coronary Arteries in Childhood Heart Disease: Implications for Management of Young Adults. J. Clin. Exp. Cardiolog..

[40] Shah S., Rajiah P. (2016). Single coronary artery with a pre-pulmonic dual left anterior descending artery and a retro-aortic left circumflex artery. Cardiol. Young.

[41] Mahle W.T., Gonzalez J.H., Kreeger J., Marx G., Duldani G., Silverman N.H. (2012). Echocardiography of Transposition of the Great Arteries. Cardiol. Young.

[42] Martins P., Castela E. (2008). Transposition of the great arteries. Orphanet J. Rare Dis..

[43] Di Salvo G., Al Bulbul Z., Issa Z., Fadel B., Al-Sehly A., Pergola V., Al Halees Z., Al Fayyadh M. (2016). Left Ventricular Mechanics after Arterial Switch Operation: A Speckle-Tracking Echocardiography Study. J. Cardiovasc. Med..

[44] Bragantini G., Bartolacelli Y., Balducci A., Ciuca C., Gesuete V., Palleri D., Egidy Assenza G., Mariucci E., Angeli E., Gargiulo G.D. (2022). Left Ventricle Function after Arterial Switch Procedure for D-Transposition of the Great Arteries: Long Term Evaluation by Speckle-Tracking Analysis. Int. J. Cardiol. Congenit. Heart Dis..

[45] Ro S.S., Wan Q., Pasumarti N., Keelan J., Shah A., Krishnamurthy G., Choudhury T.A., Anderson B.R., LaPar D., Bacha E. (2023). Post-Operative Troponin Levels and Left Ventricular Function in Patients with d-Transposition of the Great Arteries Following the Arterial Switch Operation. Int. J. Cardiovasc. Imaging.

[46] Buendía-Fuentes F., Lozano-Edo S., Jover-Pastor P., Sánchez-Martínez J.C., Martínez-Sole J., Rodríguez-Serrano M., Aguero J., Arnau-Vives M.A., Osa-Sáez A., Martínez-Dolz L.V. (2024). Left Atrial Strain in Adults after the Arterial Switch Operation for Transposition of the Great Arteries. Echocardiography.

[47] van der Palen R.L.F., van der Bom T., Dekker A., Tsonaka R., van Geloven N., Kuipers I.M., Konings T.C., Rammeloo L.A.J., Ten Harkel A.D.J., Jongbloed M.R.M. (2019). Progression of Aortic Root Dilatation and Aortic Valve Regurgitation after the Arterial Switch Operation. Heart Br. Card. Soc..

[48] Cifra B., Dragulescu A., Border W.L., Mertens L. (2015). Stress echocardiography in paediatric cardiology. Eur. Heart J. Cardiovasc. Imaging.

[49] Ermis P. (2017). Stress Echocardiography: An Overview for Use in Pediatric and Congenital Cardiology. Congenit. Heart Dis..

[50] Luijnenburg S.E. (2019). Prevalence and Predictors of Myocardial Ischemia in Patients with Transposition of the Great Arteries 25 Years after Arterial Switch Operation. Eur. Heart J. Cardiovasc. Imaging.

[51] Kumar V., Ward C., Justo R., Anderson B. (2021). The Gore Septal Occluder (GSO) for Multiple Indications in Children—An Intention to Treat Analysis. Heart Lung Circ..

[52] Silvilairat S. (2015). Speckle-Tracking Echocardiography in Pediatric Patients with Transposition of the Great Arteries: A Comparative Study with Magnetic Resonance Imaging. J. Am. Soc. Echocardiogr..

[53] Valsangiacomo Buechel E.R., Grosse-Wortmann L., Fratz S., Eichhorn J., Sarikouch S., Greil G.F., Beerbaum P., Bucciarelli- Ducci C., Bonello B., Sieverding L. (2015). Indications for cardiovascular magnetic resonance in children with congenital and acquired heart disease: An expert consensus paper of the Imaging Working Group of the AEPC and the Cardiovascular Magnetic Resonance Section of the EACVI. Eur. Heart J. Cardiovasc. Imaging.

[54] Moscatelli S., Leo I., Lisignoli V., Boyle S., Bucciarelli-Ducci C., Secinaro A., Montanaro C. (2023). Cardiovascular Magnetic Resonance from Fetal to Adult Life-Indications and Challenges: A State-of-the-Art Review. Children.

[55] Azarine A., Garçon P., Stansal A., Canepa N., Angelopoulos G., Silvera S., Sidi D., Marteau V., Zins M. (2019). Four-dimensional Flow MRI: Principles and Cardiovascular Applications. RadioGraphics.

[56] Fratz S., Chung T., Greil G.F., Samyn M.M., Taylor A.M., Buechel E.R.V., Yoo S.-J., Powell A.J. (2013). Guidelines and protocols for cardiovascular magnetic resonance in children and adults with congenital heart disease: SCMR expert consensus group on congenital heart disease. J. Cardiovasc. Magn. Reson..

[57] Jalili M.H., Yu T., Hassani C., Prosper A.E., Finn J.P., Bedayat A. (2022). Contrast-enhanced MR Angiography without Gadoliniumbased Contrast Material: Clinical Applications Using Ferumoxytol. Radiol. Cardiothorac. Imaging.

[58] Nazarian S., Beinart R., Halperin H.R. (2013). Magnetic resonance imaging and implantable devices. Circ. Arrhythmia Electrophysiol..

[59] Do C., DeAguero J., Brearley A., Trejo X., Howard T., Escobar G.P., Wagner B. (2020). Gadolinium-Based Contrast Agent Use, TheirnSafety, and Practice Evolution. Kidney360.

[60] Sun L., Lee F.-T., van Amerom J.F.P., Freud L., Jaeggi E., Macgowan C.K., Seed M. (2021). Update on fetal cardiovascular magnetic resonance and utility in congenital heart disease. J. Congenit. Heart Dis..

[61] Pozza A., Reffo E., Castaldi B., Cattapan I., Avesani M., Biffanti R., Cavaliere A., Cerutti A., Di Salvo G. (2023). Utility of Fetal Cardiac Resonance Imaging in Prenatal Clinical Practice: Current State of the Art. Diagnostics.

[62] Lloyd D.F.A., van Amerom J.F.P., Pushparajah K., Simpson J.M., Zidere V., Miller O., Sharland G., Allsop J., Fox M., Lohezic M. (2016). An exploration of the potential utility of fetal cardiovascular MRI as an adjunct to fetal echocardiography. Prenat Diagn..

[63] Porayette P., van Amerom J.F., Yoo S.-J., Jaeggi E., Macgowan C.K., Seed M. (2015). MRI shows limited mixing between systemic and pulmonary circulations in foetal transposition of the great arteries: A potential cause of in utero pulmonary vascular disease. Cardiol. Young.

[64] Panayiotou H.R., Mills L.K., Broadbent D.A., Shelley D., Scheffczik J., Olaru A.M., Jin N., Greenwood J.P., Michael H., Plein S. (2023). Comprehensive Neonatal Cardiac, Feed and Wrap, Non-contrast, Non-sedated, Free-breathing Compressed Sensing 4D Flow MRI Assessment. J. Magn. Reason. Imaging.

[65] Canan A., Ashwath R., Agarwal P.P., François C., Rajiah P. (2021). Multimodality Imaging of Transposition of the Great Arteries. Radiographics.

[66] Beek F.J.A., Beekman R.P., Dillon E.H., Mali W.P.T.M., Meiners L.C., Kramer P.P.G., Meyboom E.J. (1993). MRI of the pulmonary artery after arterial switch operation for transposition of the great arteries. Pediatr. Radiol..

[67] Gutberlet M., Boeckel T., Hosten N., Vogel M., Kühne T., Oellinger H., Ehrenstein T., Venz S., Hetzer R., Bein G. (2000). Arterial switch procedure for D-transposition of the great arteries: Quantitative midterm evaluation of hemodynamic changes with cine MR imaging and phase-shift velocity mapping-initial experience. Radiology.

[68] McConnell M.V., Ganz P., Selwyn A.P., Li W., Edelman R.R., Manning W.J. (1995). Identification of anomalous coronary arteries and their anatomic course by magnetic resonance coronary angiography. Circulation.

[69] Stagnaro N., Moscatelli S., Cheli M., Bondanza S., Marasini M., Trocchio G. (2023). Dobutamine Stress Cardiac MRI in Pediatric Patients with Suspected Coronary Artery Disease. Pediatr. Cardiol..

[70] Moscatelli S., Bianco F., Cimini A., Panebianco M., Leo I., Bucciarelli-Ducci C., Perrone M.A. (2023). The Use of Stress Cardiovascular Imaging in Pediatric Population. Children.

[71] Sampson C., Kilner P.J., Hirsch R., Rees R.S., Somerville J., Underwood S.R. (1994). Venoatrial pathways after the Mustard operation for transposition of the great arteries: Anatomic and functional MR imaging. Radiology.

[72] Groenink M., Mulder B., van der Wall E. (1999). Value of magnetic resonance imaging in functional assessment of baffle obstruction after the Mustard procedure. J. Cardiovasc. Magn. Reson..

[73] Holmqvist C., Oskarsson G., Stahlberg F., Thilen U., Bjorkhem G., Laurin S. (1999). Functional evaluation of extracardiac ventriculopulmonary conduits and of the right ventricle with magnetic resonance imaging and velocity mapping. Am. J. Cardiol..

[74] Ntsinjana H.N., Capelli C., Biglino G., Cook A.C., Tann O., Derrick G., Taylor A.M., Schievano S. (2014). 3D Morphometric Analysis of the Arterial Switch Operation Using In Vivo MRI Data. Clin. Anat..

[75] Contreras J.R., Villemain O., Marini D., Dragulescu A., Yoo S.-J., Barron D.J. (2021). Utility of a bespoke 3-dimensional printed model in complex transposition. JTCVS Tech..

[76] Sachdeva R., Valente A.M., Armstrong A.K., Cook S.C., Han B.K., Lopez L., Lui G.K., Pickard S.S., Powell A.J., Bhave N.M. (2020). ACC/AHA/ASE/HRS/ISACHD/SCAI/SCCT/SCMR/SOPE 2020 Appropriate Use Criteria for Multimodality Imaging During the Follow-Up Care of Patients with Congenital Heart Disease: A Report of the American College of Cardiology Solution Set Oversight Committee and Appropriate Use Criteria Task Force, American Heart Association, American Society of Echocardiography, Heart Rhythm Society, International Society for Adult Congenital Heart Disease, Society for Cardiovascular Angiography and Interventions, Society of Cardiovascular Computed Tomography, Society for Cardiovascular Magnetic Resonance, and Society of Pediatric Echocardiography. J. Am. Coll. Cardiol..

[77] Mortensen K.H., Tann O. (2018). Computed Tomography in Paediatric Heart Disease. Br. J. Radiol..

[78] Kumar P., Bhatia M. (2022). Role of CT in the Pre- and Postoperative Assessment of Conotruncal Anomalies. Radiol. Cardiothorac. Imaging.

[79] Han B.K., Rigsby C.K., Hlavacek A., Leipsic J., Nicol E.D., Siegel M.J., Bardo D., Abbara S., Ghoshhajra B., Lesser J.R. (2015). Computed Tomography Imaging in Patients with Congenital Heart Disease Part I: Rationale and Utility. An Expert Consensus Document of the Society of Cardiovascular Computed Tomography (SCCT): Endorsed by the Society of Pediatric Radiology (SPR) and the North American Society of Cardiac Imaging (NASCI). J. Cardiovasc. Comput. Tomogr..

[80] Young C., Taylor A.M., Owens C.M. (2011). Paediatric Cardiac Computed Tomography: A Review of Imaging Techniques and Radiation Dose Consideration. Eur. Radiol..

[81] Francone M., Gimelli A., Budde R.P.J., Caro-Dominguez P., Einstein A.J., Gutberlet M., Maurovich-Horvat P., Miller O., Nagy E., Natale L. (2022). Radiation Safety for Cardiovascular Computed Tomography Imaging in Paediatric Cardiology: A Joint Expert Consensus Document of the EACVI, ESCR, AEPC, and ESPR. Eur. Hear. J.-Cardiovasc. Imaging.

[82] Pickard S.S., Armstrong A.K., Balasubramanian S., Buddhe S., Crum K., Kong G., Lang S.M., Lee M.V., Lopez L., Natarajan S.S. (2023). Appropriateness of Cardiovascular Computed Tomography and Magnetic Resonance Imaging in Patients with Conotruncal Defects. J. Cardiovasc. Comput. Tomogr..

[83] Swanson S.K., Sayyouh M.M., Bardo D.M.E., Ghadimi Mahani M., Lu J.C., Dorfman A.L., Agarwal P.P. (2018). Interpretation and Reporting of Coronary Arteries in Transposition of the Great Arteries: Cross-Sectional Imaging Perspective. J. Thorac. Imaging.

[84] Lembcke A., Koch C., Dohmen P.M., Rutsch W., Abbara S., Krug L.D., Muehler M.R., Rogalla P. (2005). Electrocardiographic-Gated Multislice Computed Tomography for Visualization of Cardiac Morphology in Congenitally Corrected Transposition of the Great Arteries. J. Comput. Assist. Tomogr..

[85] Pergola V., Avesani M., Reffo E., Da Pozzo S., Cavaliere A., Padalino M., Vida V., Motta R., Di Salvo G. (2023). Unveiling the gothic aortic arch and cardiac mechanics: Insights from young patients after arterial switch operation for d-transposition of the great arteries. Monaldi Arch. Chest Dis..

[86] Zucker E.J. (2022). Computed Tomography in Tetralogy of Fallot: Pre- and Postoperative Imaging Evaluation. Pediatr. Radiol..

[87] Tsai-Goodman B., Geva T., Odegard K.C., Sena L.M., Powell A.J. (2004). Clinical Role, Accuracy, and Technical Aspects of Cardiovascular Magnetic Resonance Imaging in Infants. Am. J. Cardiol..

[88] Ranganath P., Singh S., Abbara S., Agarwal P.P., Rajiah P. (2019). Computed Tomography in Adult Congenital Heart Disease. Radiol. Clin. N. Am..

